# Large-scale genotypic identification reveals density-dependent natal dispersal patterns in an elusive bird of prey

**DOI:** 10.1186/s40462-023-00447-5

**Published:** 2024-02-15

**Authors:** Ida Penttinen, Carina Nebel, Torsten Stjernberg, Laura Kvist, Suvi Ponnikas, Toni Laaksonen

**Affiliations:** 1 Department of Biology, University of Turku, Turku, Finland; 2 Finnish Museum of Natural History, University of Helsinki, Helsinki, Finland; 3 Ecology and Genetics Research Unit, University of Oulu, Oulu, Finland

**Keywords:** Movement ecology, Population biology, Raptor, Microsatellites, Haliaeetus albicilla, Ecological genetics

## Abstract

**Background:**

Natal dispersal, the distance between site of birth and site of first breeding, has a fundamental role in population dynamics and species’ responses to environmental changes. Population density is considered a key driver of natal dispersal. However, few studies have been able to examine densities at both the natal and the settlement site, which is critical for understanding the role of density in dispersal. Additionally, the role of density on natal dispersal remains poorly understood in long-lived and slowly reproducing species, due to their prolonged dispersal periods and often elusive nature. We studied the natal dispersal of the white-tailed eagle (*Haliaeetus albicilla*) in response to local breeder densities. We investigated the effects of the number of active territories around the natal site on (a) natal dispersal distance and (b) the difference between natal and settlement site breeder density. We were interested in whether eagles showed tendencies of conspecific attraction (positive density-dependence) or intraspecific competition (negative density-dependence) and how this related to settlement site breeder density.

**Methods:**

We used a combination of long-term visual and genotypic identification to match individuals from their breeding site to their natal nest. We identified natal dispersal events for 355 individuals hatched between 1984 and 2015 in the Baltic Sea coast and Arctic areas of Finland. Of those, 251 were identified by their genotype.

**Results:**

Individuals born in high-density areas dispersed shorter distances than those born in low-density areas, but settled at lower density breeding sites in comparison to their natal site. Eagles born in low natal area densities dispersed farther but settled in higher density breeding sites compared to their natal site.

**Conclusions:**

We show that eagles might be attracted by conspecifics (positive density-dependence) to identify high-quality habitats or find mates, but do not settle in the most densely populated areas. This indicates that natal dispersal is affected by an interplay of conspecific attraction and intraspecific competition, which has implications for population dynamics of white-tailed eagles, but also other top predators. Furthermore, our study demonstrates the value of long-term collection of both nestling and (non-invasive) adult DNA samples, and thereafter using genotype matching to identify individuals in long-lived and elusive species.

**Supplementary Information:**

The online version contains supplementary material available at 10.1186/s40462-023-00447-5.

## Introduction

Natal dispersal, the movement of an individual animal from the site of birth to the site of first reproduction [1, 2], is a key component shaping the structure of populations, communities, and ecosystems [3–5] as it is largely responsible for gene flow and colonization of new areas [3, 4, 6]. The patterns of natal dispersal can determine the response of a species or population to the creation, degradation, or saturation of habitats. Knowledge of natal dispersal behaviour and its potential mechanisms is important for understanding eco-evolutionary processes and making more informed conservation and management actions [6, 7].

Population density is one of the key factors affecting dispersal patterns. The relationship between natal dispersal and population density can, however, be complex, as both positive and negative density-dependent mechanisms are supported by theory and empirical data [8, 9]. Positive density-dependence means that dispersal distances or rates increase with increasing population density and is commonly observed in birds and mammals [9]. It is likely a response to increased intraspecific competition, which forces maturing individuals to go farther to less dense areas to find vacant settlement sites. Negative density-dependence means that dispersal distances or rates decrease with increasing population density. It can arise e.g. if the presence of conspecifics is used as a cue for high-quality breeding sites [3, 4, 8, 9]. Distinguishing between positive and negative density-dependent dispersal or their relative roles in the distribution of individuals is challenging if densities in both natal and settlement areas are not known. It is a shortcoming of most studies on the effects of density on dispersal that they are examining emigration rates with no information on the fate of the individuals thereafter, or that they examine dispersal distances in relatively small study areas where spatial variation in density is not reported [9]. The density-dependent responses can be better understood if they are studied at the individual level in a system where the outcome of the settlement process is also considered. Thus, despite its importance in biological processes, natal dispersal is not well understood over large spatial scales because of many challenges for research [5, 10].

The traditional method for studying natal dispersal is marking young individuals at their birth site and deriving natal dispersal data from re-encounters during the breeding season (e.g., bird ringing) [11]. This requires either capturing or visually locating and identifying the breeding adults by their marks or tags. An animal group whose natal dispersal is particularly hard to study are large, long-lived, and mobile species such as large raptors, for which acquiring data on relevant spatiotemporal scales can be especially problematic [12, 13]. Many raptor species exhibit delayed maturity, which poses temporal challenges for natal dispersal studies. Especially in large species, the time before the first possible recapture at the breeding site can be very long, even up to a decade [14, 15]. While it is possible to ring or otherwise tag raptor nestlings, recapture of adults is often difficult, if not impossible. Recapture can be circumvented by the use of marks that can be observed from a distance with e.g. a telescope or a camera, enabling the identification without actual physical capture [11, 13]. However, this can still yield low recapture rates despite intense search efforts, especially when working with elusive and shy species. Most accurate movement data with high temporal and spatial resolution can be achieved with the use of telemetry. However, the battery lifespan and correct functioning of the tracking devices still limits the tracking of full dispersal process in species with prolonged dispersal periods, and the high device cost restricts the sample size [16, 17].

The use of non-invasive sampling and molecular genetic tools for identification of individuals is a valid alternative for studying elusive species, and the only alternative for species that are impossible to sample otherwise [18, 19]. Non-invasive genetic sampling allows collecting genetic material from hard-to-capture raptors without the need to capture, handle or even observe them. DNA can be extracted from e.g., feathers that raptors shed at the nest sites [19–22]. This method holds many potential benefits for conducting large-scale, non-invasive studies on raptors, and to some extent circumvents the challenges mentioned above. Since the sample collection usually does not require specialized skills or education, volunteers and citizen science can be used to increase the sampling effort, and sample collection can be made a part of the population monitoring routine. Highly polymorphic nuclear markers, such as microsatellites, have already been used in several natal dispersal studies of birds and other species [23–28]. However, these studies mostly infer natal dispersal distance or propensity indirectly from spatial genetic structures of the populations [26, 27], locations of parents and offspring based on parentage analysis [24, 28], or use assignment tests to find the population of origin [23, 25, 26]. This rarely allows assigning accurate enough local or perceived population densities to both natal and settlement sites. However, microsatellite genotypes can also be used as a direct method, if the same individual is sampled as a young at the natal nest and later as a breeding adult. This method allows the measurement of exact natal dispersal distance, especially for raptors with easily determined breeding locations (nests) and relatively high breeding site fidelity. To our knowledge, this method has thus far very rarely been used in natal dispersal studies [29].

We examined the effect of breeding density on dispersal and settlement decisions in a large and long-lived raptor, the white-tailed eagle (*Haliaeetus albicilla*, hereafter ’WTE’) by using molecular identification from samples collected at both natal and breeding nests of individuals and supplementing this data with visual identifications. The WTE is a highly mobile apex predator, which has experienced a population bottleneck due to persecution and environmental pollution [30]. In Finland, WTE almost went extinct in the 1920s and 1970s, but has recovered to over 600 breeding pairs due to effective conservation measures ([31], Osprey foundation, unpublished data). This population offers the unique possibility for a spatially and temporally large-scale study of natal dispersal and the influence of density on settlement decisions. This is due to a nationwide annual monitoring of all known territories and a ringing scheme that was initiated during the population low in the 1970s [31–33]. The collection of nestling and adult feathers for molecular genetic identification has been part of the annual monitoring since 2003. We (1) investigated the effects of natal area breeder density on natal dispersal distance of the WTE and (2) examined whether they settle to breed in areas of higher or lower density compared to the natal area. We expect to see positive density-dependence in natal dispersal distance and a higher propensity to settle in lower density areas than the natal area, if increased competition for local resources in high density areas is the key driver of dispersal. However, if conspecific attraction plays a more important role than competition, the relationships could be the other way around. The results of this study allow us to better understand the interplay between density and dispersal in expanding populations and give us insights into factors driving natal dispersal behaviour in general.

## Material & methods

### Study area and field procedures

**Fig. 1:**
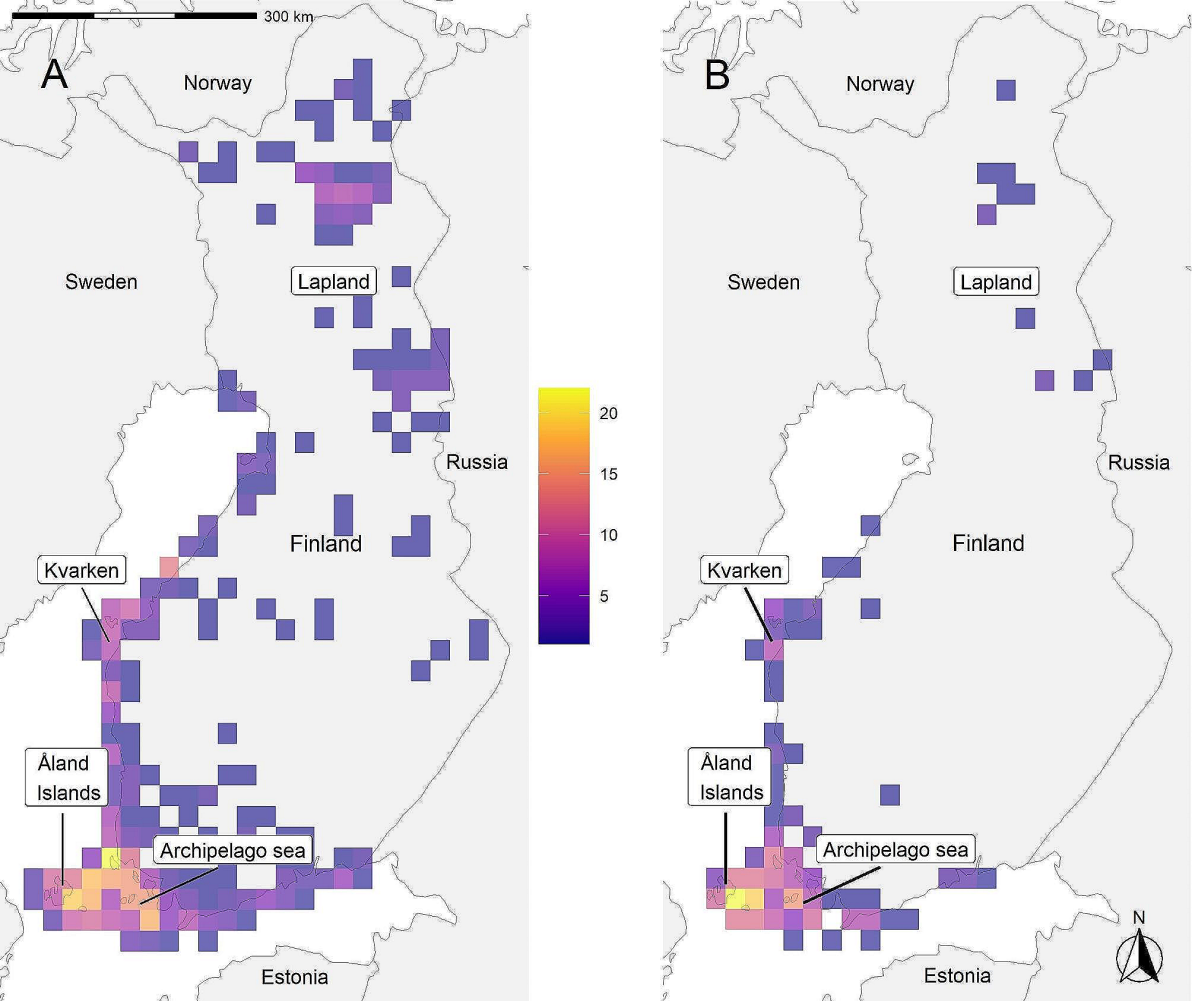
Map of the study area (Finland). Core areas of the WTE population are Archipelago Sea, Åland Islands, Kvarken area and Lapland, but new territories have been established around the country during the last few decades. Maps show (**A**) the distribution of the WTE population based on the active territories in 2022, and (**B**) observed natal dispersal events (natal territories of the individuals) in 25 × 25 km grid cells. Colors indicate the number of territories with low densities in violet and high densities in yellow

WTE is a large bird of prey, which is widespread in the northern Palearctic. They defend a small nesting territory, while the home range can be considerably larger and overlap with other WTE pairs [34]. In Finland the WTE breeds mainly along the coastline, but also in Lapland and near large inland water bodies. The core population areas are the Åland Islands, Archipelago Sea in Southwest Finland, and the Kvarken area (Fig. 1).

Every spring, volunteers of the White-tailed eagle work group (under WWF Finland from 1973 to 2019 and Osprey Foundation 2020 onwards) check the status of the known nest sites, take GPS coordinates of newly found nests, ring the nestlings, and take pictures of the parent birds for ring-reading. In Finland, WTE nestlings are ringed with one metal ring that has an engraved 5-digit individual ID code and a second color-ring with a shorter unique alphanumeric code [32] for adult identification with a camera from the distance. The sex of the parents is determined based on visual or behavioral cues. From each ringed nestling, feather samples were collected from 2003 onwards for DNA identification. To identify breeding adult eagles, we used both ring-reading and genetic identification. From 2003 onwards, the nests and their surroundings were searched for shed adult feathers for DNA identification.

### DNA extraction and genotyping

Laboratory procedures were carried out by the Center of Evolutionary Applications (CEA, University of Turku). DNA was extracted from 1519 nestling feathers (1 feather/nestling) collected between 2008 and 2015, and 2139 adult feathers collected between 2012 and 2022 (1 feather/nest/year). In addition, 784 existing nestling genotypes from years 2003–2007 [35] were used (see details below). The differing year ranges for nestling and parent genotyping were selected to allow the nestlings time to settle as breeders.

Nestling and adult feathers were prepared for DNA extraction by cutting a small piece (approx. 0.5 cm) from the basal tip of the calamus. DNA extraction from the nestling feathers was done with a silica fine and filter-based method modified from [36]. DNA from the adult feathers was extracted using a salt extraction method modified from [37]. Samples were genotyped using 14 loci [35, 38]. For the microsatellite analysis, two multiplex PCRs were used, and one locus was amplified separately (Multiplex 1: Hal-01, Hal-07, Hal-09, Hal-03, Hal-13, Aa-27, IE-12, Hal-04, Hal-05; Multiplex 2: IE-04, Hal-06, Aa-11, IE-11; single Hal-14). The samples were sexed by amplifying short diagnostic fragments of the Spindlin gene (marker Z37B) [39]. Microsatellites were amplified in 12 µl reaction volumes using QIAGEN Multiplex PCR Kit (Qiagen Inc. Valencia, CA, USA), and contained 1 µl of primer mix, 3 µl of DNA, 2 µl of MQ-H_2_O and 6 µl of QMP. Primer concentrations were 0.096 µM for all primers, except for Hal-14 and Z37B, for which a primer concentration of 0.19 µM was used following the standard protocol. To improve the microsatellite peak profiles, a GTTT-tail was added to the 5’ end of each reverse primer [40]. The following PCR profile was used: 94 °C for 15 min followed by 34 cycles of 94 °C for 30 s, annealing at 58 °C for 90 s and 72 °C for 60 s, and a final extension at 72 °C for 10 min. Amplifications were performed on Bio-Rad S1000 and Applied Biosystems 2720 thermal cyclers. PCR products from the two multiplexes and two single primer runs were pooled for the fragment analysis, which was performed by capillary electrophoresis on an ABI PrismTM 3130xl genetic analysis instrument. The peak profiles of the pooled samples could then be separated during scoring and visual inspection, using GeneMarker version 2.4.0 (SoftGenetics).

The 2003–2007 microsatellite dataset included allele sizes for 9 loci: Hal-01, Hal-03, Hal-04, Hal-05, Hal-06, Hal-07, Hal-09, Hal-13, and Hal-14. To calibrate microsatellite scoring between different laboratories, we obtained DNA profiles from 78 individuals that had been processed by [35]. Those samples were repeated at CEA lab to compare and combine the two datasets.

### Individual identification

To identify nestlings that have recruited into the adult breeding population, we screened the genotypes from adult feathers for matches in the genotypes of the nestlings with GIMLET software [41] (default settings, missing alleles treated as any allele). All available loci were used when matching the breeding adults with nestlings. When comparing the adult data with the nestling data from 2003 to 2007, the 9 overlapping loci were used. Genotypes with less than 9 loci detected were removed from the analysis.

The suitability of the two sets of loci for individual identification was determined by calculating the combined non-exclusion probability (identity) and combined non-exclusion probability (sib identity) with Cervus 3.0.7 [42].

### Natal dispersal distance and natal area breeder density

The natal dispersal distance was measured as the geodesic distance (km) between coordinates of the natal nest and the nest where the same individual was detected for the first time as a breeding adult. Breeding dispersal is very rare in the white-tailed eagle [43], and thus it is safe to assume that the site of first detection is probably the site of first breeding attempt. The natal area breeder density was measured as the number of active territories within 30 km radius of the natal nest in the year of expected recruitment [31]. We used the fixed year of expected recruitment (natal year + 5) for the density calculations, since we considered it the best single descriptor from the eagle’s point of view. At this time, the WTEs have reached sexual maturity and it can be assumed that they started looking for a mate and territory. While it would be ideal to use the actual year of settlement, the year of first detection may not be coinciding with the first breeding attempt, despite the high monitoring efforts of our population. Eagles are likely to prospect potential breeding sites several years before settling, but the local densities in this population are not changing that much in consecutive years that it would be biologically meaningful to test for these effects.

The 30 km radius was chosen based on the median natal dispersal distances found in this study (see results below). Enlarging the buffer from 30 km did not give us any additional data, since larger buffers likely include more areas that are not inhabited by white-tailed eagles e.g., open sea. The natal area breeder densities in 30 km radius ranged from 0 to 68 territories with a mean ± SD of 31.6 ± 17.6. A territory was determined to be active if signs of occupancy were detected at the nest (at least some new nest material that has been brought in). If a territory had more than one alternative nest site, the nest showing the furthest advanced signs of occupancy or nesting (from some new nest material to ringed nestlings) was used in the analysis.

For examining the relationship between natal area breeder density and the density difference between the breeding and the natal site, we calculated the number of active territories within 10 km buffer for both the natal and the breeding site in the expected recruitment year, and subtracted the natal site density from the breeding site density. A negative value indicates that an eagle has settled in a lower density site compared to its natal site, whereas a positive value indicates that the individual has settled in a higher density site. A zero value indicates that natal and breeding sites have the same breeder densities. Since this model reflects the situation where the settlement decision has already been made, i.e., the outcome of the settlement process, we used smaller, 10 km buffer, based on maximum mean home ranges for territorial adults from radio-tracking studies (e.g. [44]). In the 10 km radius, the density values ranged from 0 to 14 with a mean ± SD of 4.2 ± 3.4. There is a possibility that growing natal site density increases the probability of settling in lower density area by chance, thus leading to an inverse relationship between natal site density and density difference between natal and breeding sites. Thus, we added expected density difference to the model as a control variable, which accounts for the average level of density in the natal area. For each individual we extracted the active territories within the actual observed natal dispersal distance from their natal nest. The density within 10 km radius was calculated for each of these active territories. The individual’s natal site density (10 km buffer) was then subtracted from the average of these densities to obtain the expected difference.

### Statistical analysis

We used a linear mixed model (LMM) to explore how natal dispersal distance (response variable, log-transformed) was affected by natal area breeder density (at 30 km radius, continuous), sex (categorical, male or female) and the year of expected recruitment (continuous).First order interactions between all explanatory variables were included in the initial models and removed when not significant. In a second LMM, the difference between breeding and natal area density (at 10 km radius) was the response variable, and natal area breeder density and expected recruitment year were the explanatory variables. Expected density difference was included as a control variable. The model was checked for multicollinearity (VIFs = 1.02–2.32). All continuous predictor variables were scaled and natal territory ID and expected recruitment year were set as crossed random effects with random intercepts in both models to account for potential non-independence of observations from the same year and same territory. Model residuals were visually inspected for normal distribution.

All statistical analyses were carried out in R version 4.1.3 [45]. Data wrangling and data visualization was done with the ‘tidyverse’ package [46]. Maps were obtained through ‘rnaturalearth’ [47] and ‘rnaturalearthdata’ packages [48]. To calculate distances between nest sites, we used the ‘geosphere’ package [49] and to obtain the number of territories in specific radii, the ‘sf’ package [50]. LMMs were fit with the ‘lme4’ package [51], and effect sizes were obtained using the ‘ggeffects’ package [52].

### Accounting for unequal recruitment probability

A common issue with natal dispersal studies is the underrepresentation of long-distance dispersers. This can also create issues with research questions if particular groups are more likely to disperse over longer distances than others. We attempted to control this bias by considering recruitment probability within the study area. We first calculated for each nestling that was ringed between 1984 and 2015, the minimum distance (in km) between its natal nest and closest inland border or non-Finnish coastline (Åland Islands and West coastal areas: Sweden; South coastal areas: Estonia; Lapland: Sweden, Norway, and Russia; Eastern inland: Russia; Fig. 1). We predicted higher emigration with closer proximity to a foreign border or coastline, resulting in loss of observations. We constructed a generalized linear model (GLM) of the binomial family, in which recruitment within the study area (0, 1) was the response variable, and the distance to the border or coastline the key explanatory variable. We found that probability of recruitment significantly increased with a larger distance to a foreign border or coastline (estimate = 0.004, SE = 0.001, df = 1, χ2 = 8.39, *p* = 0.004). We extracted predicted recruitment probability values using the ‘ggeffects’ package (mean = 0.055, SD = 0.009) [52]. Those predicted values were used as a weight in the natal dispersal model described above to adjust for the expected recruitment probability within the study area.

## Results

The combination of molecular genotyping of feathers and identification of rings from photographs was successful in linking natal and breeding site locations, as we were able to do this for 335 individuals hatched between 1984 and 2015. This data consisted of 156 females, 141 males, and 38 individuals of unknown sex. 84 individuals were identified based on ring re-sightings from photographs, 233 individuals were identified based on matching the genotypes of nestlings and adults, and 18 individuals were identified with both methods. The combined non-exclusion probability values confirmed that the set of loci used was reliable for the identification of individuals, even with the smaller set of 9 loci (Table S1). The final dataset used in the statistical analyses consisted of 285 individuals, after the exclusion of individuals of unknown sex (*n* = 38) and of age < 5 calendar years (*n* = 12).

**Fig. 2:**
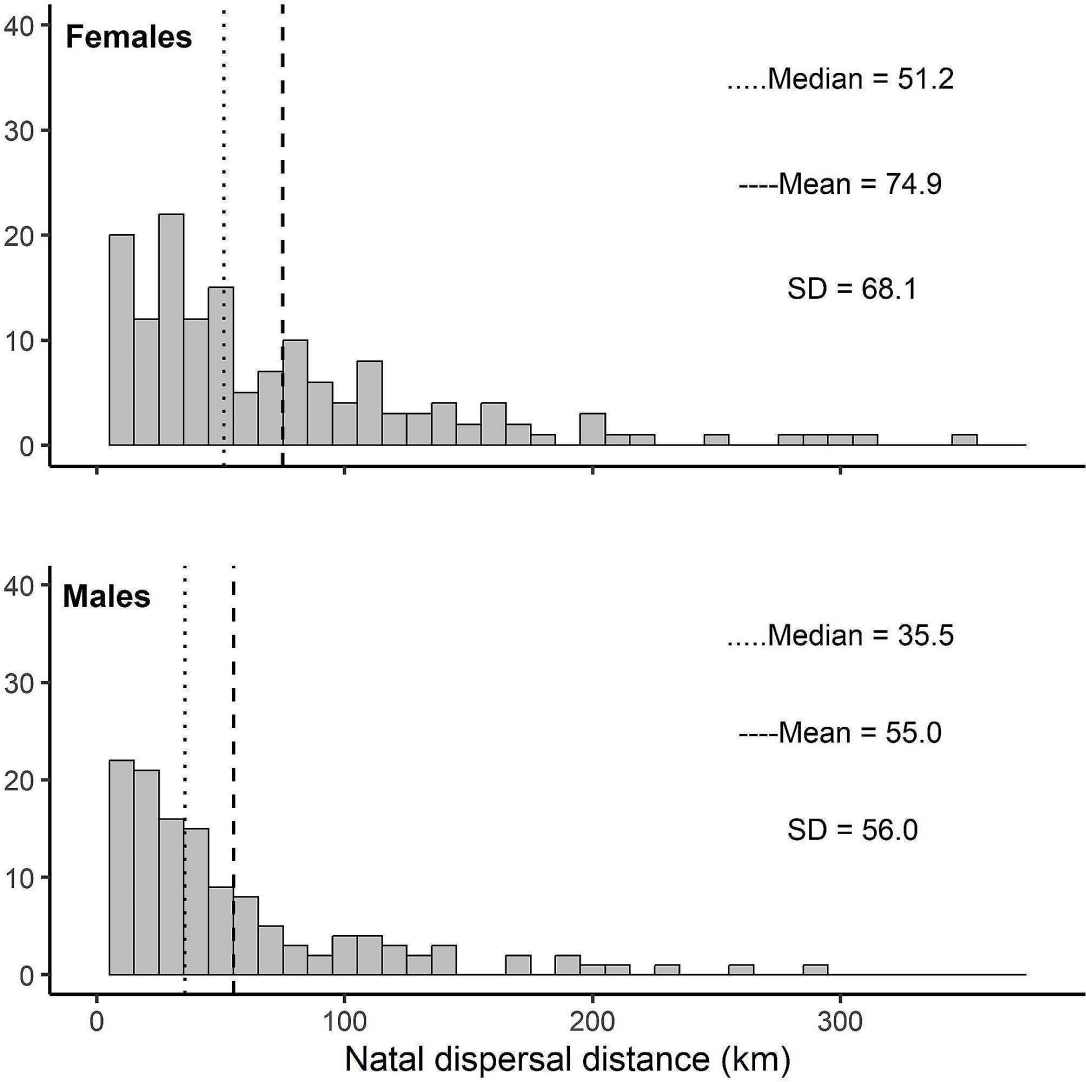
Histogram of the natal dispersal distance (km) of Finnish white-tailed eagles hatched between 1984 and 2015. Top shows natal dispersal distances for females (*n* = 156), bottom for males (*n* = 141). Dotted line indicates median, dashed line mean natal dispersal distance. Females disperse slightly farther than males. The natal dispersal distance is left-skewed for both sexes, indicative of philopatric settlement behaviour. The natal dispersal distances found in the Finnish white-tailed eagle ranged from 4.5 to 352.3 km for females and from 2.4 to 289.3 km for males

**Fig. 3:**
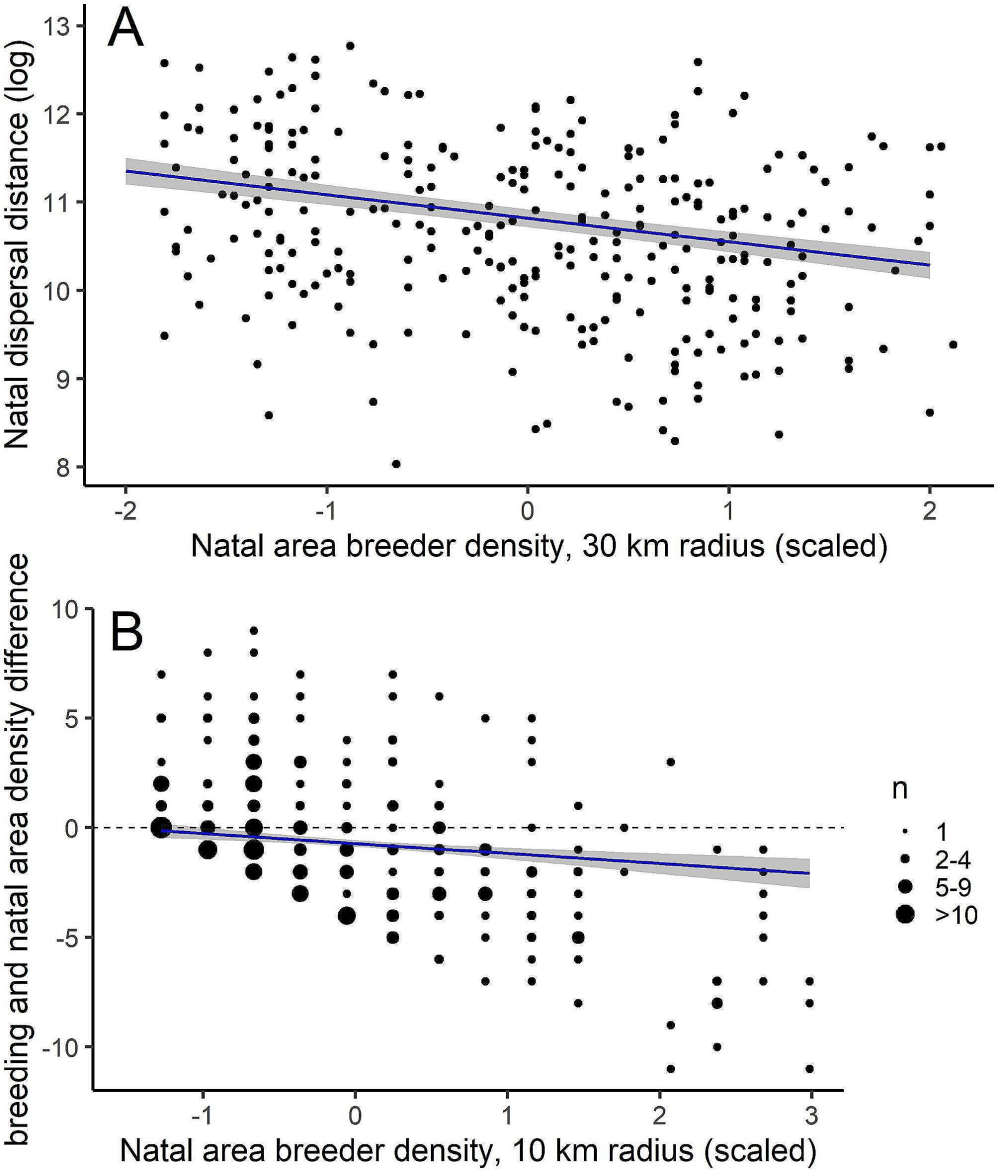
Relationship between (**A**) natal area breeder density at 30 km radius from the nest and natal dispersal distance of WTE, and (**B**) natal area breeder density and difference between breeding and natal area densities at 10 km radius at the expected recruitment year. Regression line depicts model predictions and grey areas standard errors. Log-transformed values of natal dispersal distance and scaled values of densities are shown

Natal dispersal distances ranged from 2.4 to 352.3 km (both sexes, median = 43.1 km, mean = 65.7 km ± 63.4 SD), with females dispersing significantly farther (range = 4.52–352.3, median = 51.2, mean = 74,9 km ± 68.1 SD) than males (range = 3.08–289.3 km, median = 35.5 km, mean = 55 km ± 56 SD, Fig. 2; Table 1). Natal dispersal distances were shorter with increasing natal area breeder density (Table 1; Fig. 3), and did not change during the observation time. Natal area breeder density significantly affected whether WTEs settled on a breeding site of lower or higher density compared to their natal site, even after accounting for the expected average difference within the dispersal range (Table 2). WTEs born in a low natal area density settled in breeding sites of relatively higher density, while individuals born in high natal breeder densities settled in less dense areas compared to their natal one (Table 2; Fig. 3). To interpret the above results and to verify they were not confounded by time-effects, we also analysed natal site breeder density at 10 and 30 km radii against time (expected recruitment year) but found no significant correlations (*r* = 0,058, *p* = 0.33 for 10 km, *r* = 0,11, *p* = 0.07 for 30 km, *n* = 282).

**Table 1:** Parameter estimates of the natal dispersal distance model based on 285 settlement events observed in Finnish white-tailed eagles. The response variable was natal dispersal distance (log-transformed). Explanatory variables fitted were sex (factor, ‘male’ or ‘female’), density around the natal nest in a 30 km radius (numeric, range 0–68), and year of expected recruitment (numeric, 1989–2022). Estimates, SE, χ2, df (Satterthwaite’s) and *p*-values of main fixed effects are shown. Natal territory ID and year of expected recruitment (categorical) were set as random effects in the model. All numeric predictors were scaled and statistically significant values are highlighted in bold

Explanatory variables	Estimate	SE	χ^2^	df	*p*
**Intercept**	**10.821**	**0.099**	**11932.61**	**26.36**	**< 0.0001**
**Sex (male)**	**-0.394**	**0.108**	**13.20**	**269.94**	**0.0003**
**Natal area breeder density**	**-0.267**	**0.057**	**22.14**	**182.73**	**< 0.0001**
Expected recruitment year	-0.065	0.075	0.74	50.65	0.3941
Random effects	Variance	SD	n		
Natal territory ID	0.065	0.255	180		
Expected recruitment year	0.067	0.259	24		

**Table 2:** Parameter estimates of the density difference model based on 285 settlement events observed in Finnish white-tailed eagles. The response variable was the difference between breeding and natal area densities at 10 km radius from the nests. Explanatory variables fitted were density around the natal nest in a 10 km radius (numeric, range 0–14), and expected recruitment year (numeric, 1989–2022). Expected density difference (numeric, range − 7–9) was included as a control variable. Estimates, SE, χ2, df (Satterthwaite’s) and *p*-values of fixed effects are shown. Variance and SD are shown for random effects, natal territory ID and expected recruitment year. All numeric predictors were scaled and statistically significant values are highlighted in bold

Explanatory variables	Estimate	SE	χ^2^	df	*p*
**Intercept**	**-0.718**	**0.148**	**23.56**	**278**	**< 0.0001**
**Natal area breeder density**	**-0.459**	**0.221**	**4.30**	**278**	**0.0390**
Expected recruitment year	0.147	0.165	0.80	278	0.3727
Expected density difference	1.955	0.225	75.70	278	< 0.0001
Random effects	Variance	SD	n		
Natal territory ID	0.0	0.0	179		
Expected recruitment year	0.0	0.0	24		

## Discussion

By using a combination of genetic identification and traditional bird ringing we revealed that density-dependence plays an important role in shaping natal dispersal of WTEs. We showed that WTEs return to breed close to their natal area, even though the range of natal dispersal distances can be far, up to 300 km from the natal site. We found indications that WTEs settle in higher density areas than expected in comparison to their natal area when they originate from relatively low-density areas, as opposed to the highest-density areas, from where the eagles will settle in a lower density area. WTEs born in high-density areas, however, showed the strongest philopatry and dispersed over shorter distances than those originating from a low-density natal site. Even though it is impossible for the density difference to be negative, if the natal site density is zero, there is much variation in local breeder densities (Fig. S2) and for most individuals it has been possible to choose a breeding site of relatively lower or higher density.

### Density-dependent dispersal

Conspecific attraction and intraspecific competition are considered the main drivers of density-dependence in natal dispersal, the first one causing a negative and the latter a positive relationship. In WTE we found indication for both negative and positive density dependency, but both being context-dependent: Following positive density-dependent patterns, WTEs appear to settle in higher density areas when their natal site is in a low-density area. This might be in line with the conspecific attraction hypothesis, meaning that eagles can use social information to identify high-quality sites [53]. A suitable breeding site must be first detected and then evaluated, and the presence or breeding performance of conspecifics can reduce the costs of both, increasing the detectability of the site (reduced search costs) or indicating the quality of the site (reduced evaluation costs). However, WTEs are also territorial and they regularly engage in territorial fights that can decrease survival [54–56]. At low densities, this intraspecific competition might occur less frequently than at higher densities. In line with negative density-dependency, we found indication that areas of the highest density of conspecifics might be keeping new WTEs from settling there, causing them to settle in relatively lower density areas instead. However, the rather short dispersal distances from high-density territories indicate that those individuals move short distances, i.e. to the edge of high-density areas or into pockets of lower breeder density in-between high-density areas rather than disperse over large distances. It has also been speculated that one of the reasons behind negative density-dependence in raptor dispersal is a floater strategy, in which individuals postpone the start of breeding to wait for good territories to fall vacant, instead of dispersing large distances or settling for a worse territory option [57, 58].

Between 1989 and 2020 (the range of expected recruitment years in our data), the number of known active territories in Finland increased from about 70 to well over 600 (Osprey Foundation, unpublished data). Although we show a clear relationship between breeder density and natal dispersal distance, there was no temporal trend in dispersal distances (i.e., dispersal distances becoming shorter over time, Fig. S1). It seems that although the WTE population has grown considerably, the perceived population density has not grown much on the local scale. Instead, WTE might have occurred in high densities at specific hotspots and expanded their ranges continuously from there, leading to an increased variation in local breeder densities (Fig. S2) and an overall consistent natal dispersal behaviour in relation to local breeder densities over time. This might also indicate that enough suitable habitats have been available, and the population has not reached a saturation point yet, at least not in all areas.

Other studies of density-dependent dispersal have mostly focused on how the population density at the natal site affects the natal dispersal rates or distances [9, 59, 60, 61, 62], while the relationship between natal and settlement area densities has rarely been examined. Our results demonstrate that there is a risk of misinterpretation if the settlement site density is not considered. In the present study, looking only at the effects of natal area breeder density on the natal dispersal distance would give the impression that conspecific attraction coupled with a tendency to return to natal area has a strong role in WTE natal dispersal. The inclusion of settlement site density reveals that the WTE is not unambiguously favoring settlement into areas of high conspecific density. In the relationship between density and natal dispersal distance, this is probably masked by other factors (e.g., habitat quality).

High habitat quality in natal areas can be associated with shorter dispersal distances [60, 62], and it has previously been shown that the home range sizes of the WTE are smaller in optimal habitats [34], meaning that they can support more WTEs. In such habitats, finding a good territory with less competition may not require moving as far as in lower quality habitats. Therefore, the negative relationship between density and natal dispersal distance could be due to high quality habitats being available at shorter distances for eagles that want to settle. However, when breeder density reaches a negative cost-benefit threshold due to increasing territorial disputes, moving to an area with less competition becomes the more beneficial option, which could subsequently lead to a positive density-dependent pattern [63, 64]. As the characteristics and quality of the habitat are beyond the scope of the present study, more analyses would be needed to confirm whether habitat quality is behind the observed patterns.

Previous studies have produced variable results of density-dependence in raptor dispersal, indicating positive [60, 65], negative [66, 67], or no density-dependence [68]. Studies showing that the effects of population density depend on physical condition or competitive ability of the individual, indicate that intraspecific competition is a driver of natal dispersal [66]. A similar negative relationship between population density and dispersal distance to our study has been found in other raptors [69, 66, 67] including large raptors with delayed maturity [61, 70]. This pattern is often explained by conspecific attraction and the use of density as a cue for a high-quality habitat. A positive association between population density and habitat quality has been found in Bonelli’s eagles (*Aquila fasciata*, [71]) but evidence of conspecific attraction in other species of eagles is scarce. In Bonelli’s eagles, inexperienced individuals were most attracted to previously occupied territories in areas with high breeder density. The attraction towards higher densities can also be due to prospecting of mates. This has been shown in translocated sub-adult Spanish imperial eagles (*Aquila adalberti*), which despite an advantageous competitive environment, left the translocation area to explore breeding populations over 100 km away [72].

### Distribution of natal dispersal distances

Our results indicate that the distribution of natal dispersal distances is strongly skewed towards shorter distances and that the occurrence of long-distance dispersal is relatively rare. Similar patterns have been found in numerous natal dispersal studies in raptors [69, 73–75] and other species [59, 76–78]. WTE can move over large distances, and we detected natal dispersal distances over 300 km within Finland. Individuals are lost when the study area is limited, even if the study area covers an entire country. Eagles born in the coast of Finland, especially on the Åland Islands, can find suitable breeding territories on e.g., the Swedish side of the Baltic Sea without moving longer than average distances. Therefore, not only long-distance dispersers are lost, but dispersers across all scales. As a result, we conclude that the dispersal distance distribution presented in this study gives a fairly accurate reflection of dispersal behavior and distances of WTE.

### Genotype matching as a method for measuring natal dispersal

The use of genetic methods to indirectly infer natal dispersal or combining genetic methods with mark-recapture methods has been fairly common, but using genotype matching to directly measure the natal dispersal distance is rarer [29]. Despite the challenges related to this method, our results show that it can be a useful tool for measuring natal dispersal.

The use of non-invasive sampling substantially increases re-encounter rates. Genotypes provided more than double the number of identifications (244 vs. 101) in less than half the time (14 vs. 33 years) compared to visual identifications. WTE has a high breeding site fidelity, which means that events of breeding dispersal do not confound the measurements of natal dispersal distance, even if the sample is not collected on the first breeding year. Samples collected in the vicinity of active nests belong with high certainty to the occupant of the given nest. Even though there is a possibility of finding feathers of territory intruders [79], those are mostly immature WTEs [80], and genotype matching allows exclusion of too young individuals from the analysis. Because of the long-term sampling of both chicks and adults, we were able to get multiple DNA identifications of same individuals from the same nest site in different years. This considerably increases the certainty that our identified individuals are the occupants of the sampled nests.

Genotype matching requires that an individual has been sampled as a nestling and as a breeding adult. This means that to receive enough data, the sample size must be substantial. A temporal challenge in data collection can arise when the time between the onset of natal dispersal and start of breeding is long, as it is for WTE. However, since many raptors are of conservation concern, and therefore monitored, sample collection can be made part of the monitoring routine, to ensure long-term sampling. Otherwise, genotype matching might be better suited for species with no or shorter delayed maturity.

### Conclusions & implications

In this study, we demonstrated that natal dispersal behaviour is density-dependent in the Finnish WTE. Growing breeder densities in natal areas decrease the natal dispersal distances, but at the same time, individuals born in high-density areas are settling in lower density areas compared to their natal area. With long-term sampling and genotype matching, we were able to consider the breeder densities in both the natal and breeding sites of the individuals. This revealed that the density-dependence in WTE natal dispersal is not a simple matter of intraspecific competition or conspecific attraction, but likely an interplay of the two.

Furthermore, from a conservation or management perspective, our results indicate that the study population, despite having grown extensively in the past decades, has not reached a saturation point. Insights from this study will aid in developing population growth models for the Finnish and other Northern WTE populations, and better our understanding of the species‘ population recovery in Europe.

While our results on density-dependency are mostly in line with other studies of natal dispersal in large raptors, the inclusion of settlement site density reveals novel insights on the complexity of the process. The role of other contributing factors, such as habitat and prospecting behaviour will be addressed in future studies.

### Electronic supplementary material

Below is the link to the electronic supplementary material.

Supplementary Material 1

## Data Availability

Data is available in: https://doi.org/10.6084/m9.figshare.23591850.v1. Shared data includes variables needed to reproduce the analyses. White-tailed eagles are a sensitive species, locations of territories and nest sites will not be released.

## Abbreviations

WTE: White-Tailed Eagle
