# Supplementary material for: Large-scale genotypic identification reveals density-dependent natal dispersal patterns in an elusive bird of prey
Source: Mov Ecol. 2024 Feb 15;12:16. doi: 10.1186/s40462-023-00447-5 (PMC10870540; doi:10.1186/s40462-023-00447-5)
Supplement: Supplementary file 1 — Supplementary Material 1 [file 40462_2023_447_MOESM1_ESM.docx]

Supplementary material of

**Large-scale genotypic identification reveals density-dependent natal dispersal patterns in an elusive bird of prey**

Table S1: The reliability of the set of loci used to identify WTE individuals. Combined non-exclusion probability (identity) means the average probability that a set of loci will fail differentiate between two randomly-selected individuals. Combined non-exclusion probability (sib) means average probability that a set of loci will fail differentiate between two randomly-selected full siblings.

| Number of loci | 13 | 9 |
| --- | --- | --- |
| Combined non-exclusion probability (identity) | <0.0001 | <0.0001 |
| Combined non-exclusion probability (sib) | 0.0003 | 0.0030 |

**
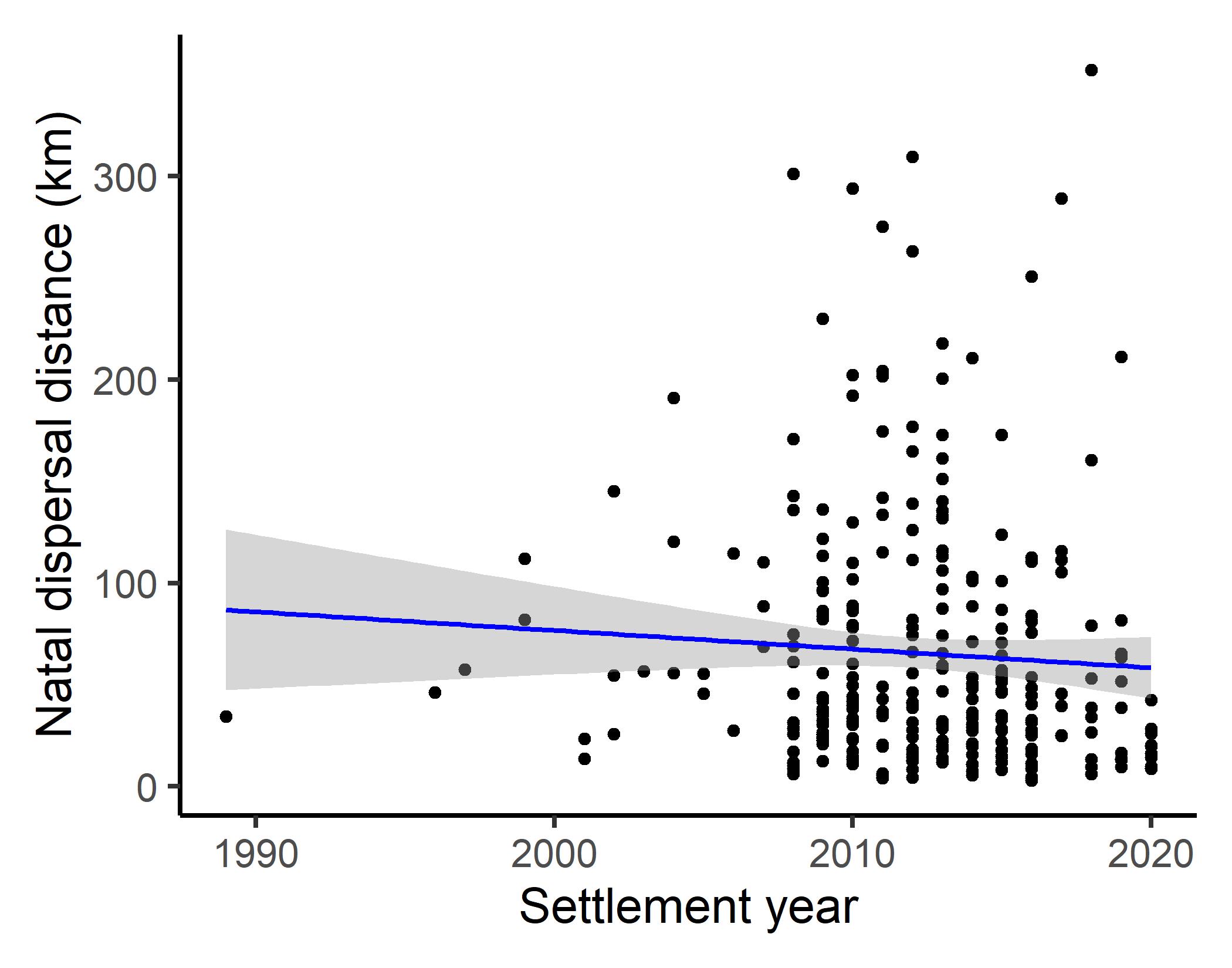
**

Figure S1: Raw data graph of the relationship between year and natal dispersal distance in kilometres.

**
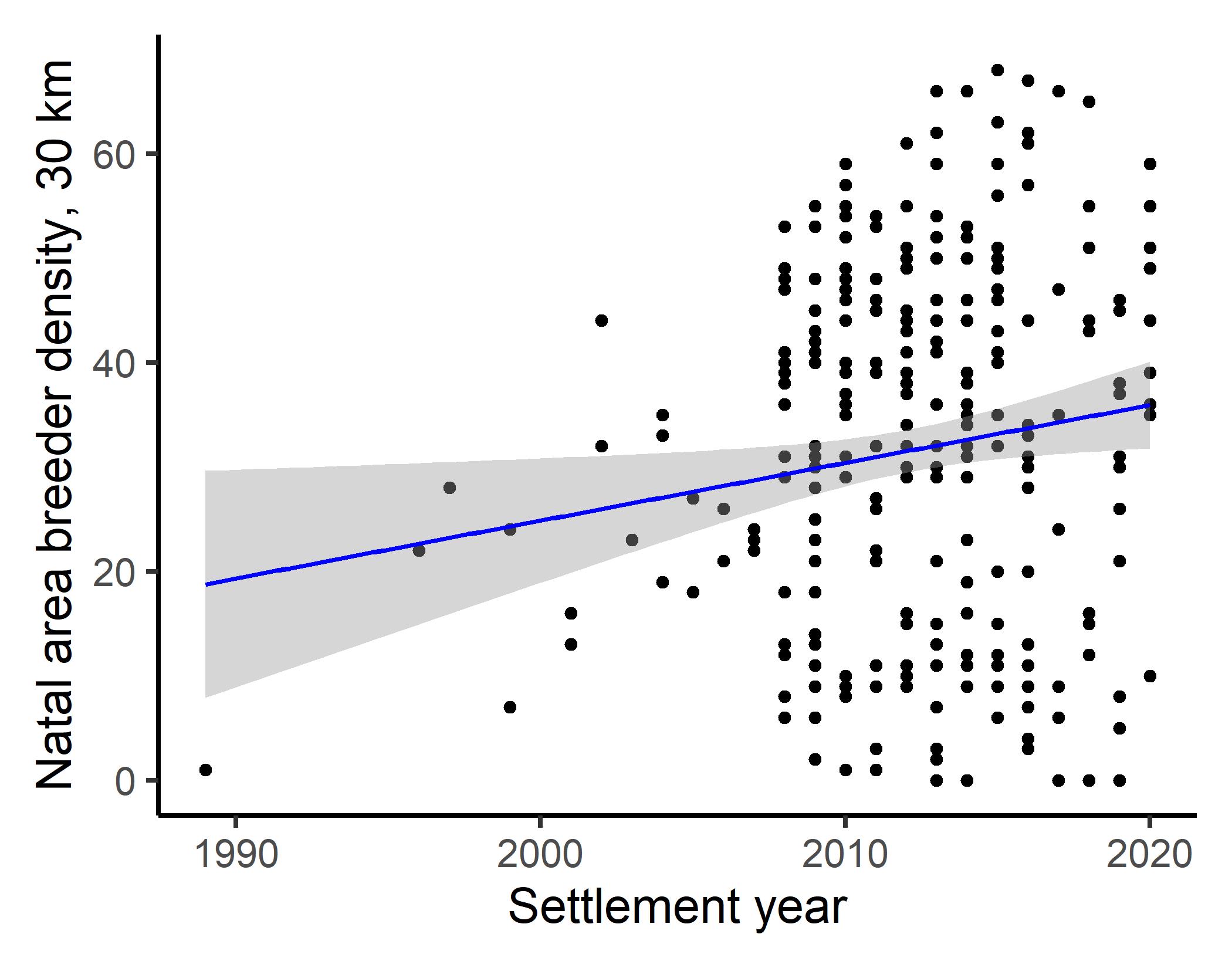
**

Figure S2: Raw data graph of the relationship between year and natal area breeder density in 30 km radius.
